# Effect of Green Tea (*Camellia sinensis*) Extract on Growth Performance, Intestinal Health, and Immune Response of Broiler Chickens During Subclinical Necrotic Enteritis

**DOI:** 10.3390/pathogens14030260

**Published:** 2025-03-06

**Authors:** Tunde E. Ogundare, Raveendra R. Kulkarni, Paul C. Omaliko, Odinaka C. Iwuozo, Ikenna G. Enenya, Oluteru E. Orimaye, Safiu A. Suberu, Olusola Jeje, Yewande O. Fasina

**Affiliations:** 1 Department of Animal Sciences, North Carolina A&T State University, Greensboro, NC 27411, USA; teogundare@aggies.ncat.edu (T.E.O.); ociwuozo@aggies.ncat.edu (O.C.I.); oeorimaye@aggies.ncat.edu (O.E.O.); ojeje@aggies.ncat.edu (O.J.); 2 Department of Population Health and Pathobiology, College of Veterinary Medicine, North Carolina State University, Raleigh, NC 27695, USA; ravi_kulkarni@ncsu.edu

**Keywords:** green tea extract, subclinical necrotic enteritis, *Clostridium perfringens*, broiler chickens

## Abstract

This study evaluated the effects of varying levels of dietary green tea extract (**GTE**) on growth performance, *Clostridium perfringens* (**CP**) colonization, and inflammatory responses in broiler chickens during experimental subclinical necrotic enteritis (**SNE**). In a 21-day experiment, 480 one-day-old male broiler (Ross 708) chicks were equally allotted into four dietary treatment groups. From days 1 to 10, all groups received a corn–soy basal diet, switching to a wheat-fish diet on day 11. Treatments included CON (no GTE), GTX, GTY, and GTZ (250, 500, and 1000 mg/kg GTE, respectively). On day 11, each group split into ACON, AGTX, AGTY, and AGTZ, totaling eight treatments. From days 17 to 20, ACON, AGTX, AGTY, and AGTZ chicks were orally challenged twice daily with 3 mL CP26 (2.5 × 10^8^ CFU/mL). On d 16 (pre-challenge) and d 1 post-challenge, ileo-jejunal contents were collected for CP enumeration, and jejunum tissue was analyzed via qRT-PCR to determine the expression levels of IL-1β, IFNγ, TNF-α, TGFβ, IL-10, and MUC2 genes. Challenged chicks showed poorer (*p* ≤ 0.05) growth and higher intestinal CP, with a potential improvement in GTE-supplemented diets. Findings suggests that dietary GTE supplementation mitigated the characteristic growth depression during SNE, reduced intestinal *CP* infection, and modulated inflammatory response in broiler chicks.

## 1. Introduction

Necrotic enteritis (**NE**) is an enteric disease in poultry caused by *Clostridium perfringens*, a rod-shaped, anaerobic, spore-forming, Gram-positive bacterium. The global poultry industry incurs an estimated annual loss of $6 billion due to NE [1,2]. *C. perfringens* is typically a component of the commensal gut microbiota; however, various predisposing factors, including coccidia infection, high stocking density, immunosuppression, gut dysbiosis, and dietary influences such as high-fish meals and non-starch polysaccharide diets, can trigger the rapid proliferation of *C. perfringens* and production of toxins such as TpeL, α-toxin, β-toxin, and necrotic enteritis B-like (NetB) toxins [1,3], leading to the onset of NE [4,5,6].

Necrotic enteritis typically affects broiler chickens aged 2 to 6 weeks and can be classified into acute clinical NE and subclinical NE (**SNE**) [7]. Acute clinical NE is characterized by diarrhea, bloody feces, intestinal ulceration, and mortality rates that can reach up to 50% [6,7]. In contrast, SNE is often asymptomatic, with low mortality rates, but generally manifests as a reduction in overall growth performance due to subtle epithelial damage that impairs nutrient absorption [6,8]. Consequently, SNE is difficult to diagnose and control promptly, leading to more widespread infections and greater economic losses than acute clinical NE [9,10].

Antimicrobial growth promoters (**AGPs**) were an effective strategy used in the past to improve production performance and control NE incidence in poultry. However, their uncontrolled use has led to the development of antimicrobial-resistant bacteria and residue accumulation, which compromise human and animal health, with its potential to threaten food safety and public health [1,11,12]. This led to the ban of AGPs in poultry production, especially the antimicrobials that are critical to human medicine [1,13]. The ban on AGPs has coincided with poor production performance and increased disease incidence [14,15], prompting the search for viable alternatives.

Recent studies have shown that NE incidence can be controlled through the administration of alternatives to AGPs [1,13,14,15,16]. Green tea extract (**GTE**) has been identified as a potential viable alternative to in-feed antibiotics in a poultry diet [17,18]. It contains polyphenols known as catechins, consisting primarily of epicatechin, gallocatechin, epigallocatechin, gallocatechin gallate, epicatechin gallate, and epigallocatechin gallate [19,20]. These catechins have been found to have antimicrobial, antioxidant, immunostimulatory and healthy microbiome promoting properties [18,21,22]. Catechins from green tea exhibit strong antibacterial activity, particularly against Gram-positive bacteria, likely due to their more susceptible cell wall structure compared to Gram-negative bacteria [23]. Epigallocatechin gallate, in particular, has a high affinity for bacterial cell wall components, potentially compromising structural integrity and inducing cell lysis [24]. Additionally, green tea polyphenols can disrupt bacterial metabolism by inhibiting key enzymes essential for nucleic acid synthesis [25]. This multifaceted mechanism not only impairs bacterial growth but also reduces virulence factors associated with pathogenicity [25]. Also, GTE exclusively contains L-theanine, an amino acid which improves growth performance, antioxidant status and promotes beneficial microbiota in broiler chickens [22].

While GTE and its polyphenols have been reported to have antibacterial activities and inhibit toxins associated with NE in vitro [17,26], its potential as an alternative to antibiotics in the control of NE incidence in poultry is still unknown. We hypothesized that optimum level of dietary GTE will mitigate SNE in broilers. This study aims to evaluate the effects of graded dietary GTE levels on growth performance, intestinal *C. perfringens* colonization and inflammatory responses in broiler chicks during experimentally induced SNE disease.

## 2. Materials and Methods

The procedures utilized in this study were approved by the Institutional Animal Care and Use Committee (IACUC) at North Carolina Agricultural and Technical State University.

### 2.1. Experimental Animals and Experimental Design

One-day-old (n = 480) Ross 708 male broiler chicks were obtained from a commercial hatchery where chicks were vaccinated against coccidiosis, infectious bronchitis virus, and Newcastle disease. Chicks were randomly allocated into four dietary treatment groups, with eight replicates pens housing 15 chicks each, in a completely randomized design. All four treatment groups were fed a corn–soybean meal (SBM) basal diet from day 1 to 10 and subsequently switched to a wheat-SBM-fish meal (high protein) diet until the end of the experiment (day 21). The four dietary treatments groups comprise Treatment 1—Control (CON), in which diets contained no green tea extract (GTE) supplementation; Treatment 2 (GTX), containing a 250 mg GTE/kg diet; Treatment 3 (GTY), containing a 500 mg GTE/kg diet; and Treatment 4 (GTZ), containing a 1000 mg GTE/kg diet. On d 11 of the experiment, each of the four treatment groups was further divided into two treatments, thereby giving non-challenged (CON, GTX, GTY, and GTZ) and corresponding CP-challenged groups ACON, AGTX, AGTY, and AGTZ, respectively. Accordingly, from d 11 to the end of the experiment (d 21), we had a total of eight treatments, each consisting of four replicates in a 2 × 4 factorial design.

The GTE used in this study was prepared from plant leaves, and obtained from Sabinsa Corporation (East Windsor, NJ, USA). It contained a total of 76.71% *w*/*w* catechins as identified by High Performance Liquid Chromatography. The identified catechins are epigallocatechin (9.10% *w*/*w*), epicatechin (5.02% *w*/*w*), epigallocatechin gallate (38.89% *w*/*w*), epicatechin gallate (11.91% *w*/*w*), catechin (1.82% *w*/*w*), and gallocatechin gallate (9.98% *w*/*w*).

#### 2.1.1. Induction of Subclinical Necrotic Enteritis

To induce SNE, basal diets were switched on d 11 to a wheat–SBM–fish meal-based high-protein (30% crude protein) diet [27] until the end of the experiment on day 21. The GTE supplementation remained the same for all treatments (Table 1). On days 17, 18, 19, and 20, chicks in the challenged treatments were orally inoculated with 3 mL of *Clostridium perfringens* (CP26 strain) isolate (about 2.5 × 10^8^ to 4.9 × 10^8^ colony-forming units (CFUs)/mL) twice daily, while those in the non-challenged treatments were mock-challenged with sterile fluid thioglycolate media.

#### 2.1.2. Growth Performance

The body weight (BW), body weight gain (BWG), and feed intake (FI) of chicks were recorded at 7, 14, and 21 days of age to evaluate broiler growth performance. From these data, the feed conversion ratio (FCR) was calculated. Mortality rates were also monitored and recorded daily throughout the 21-day experiment. The FI, BWG, and FCR during week 3 (d 14–21) were calculated to determine the growth performance during the challenge phase.

### 2.2. Sample Collection

#### 2.2.1. Sample Collection for Downstream Analyses

One chick per pen was sampled on d 16 (1 d pre-challenge) and two chicks per pen were sampled on d 21 (i.e., at 1 d post-challenge), totaling 8 chicks per treatments for both sampling days. Chicks were euthanized by CO_2_ asphyxiation.

Thereafter, the intestine of each chick was aseptically excised, and a 15 cm long ileo-jejunal segment was removed, placed in a Whirl-Pak sample bag (Nasco, Fort Atkinson, WI, USA), and kept on ice until time for transportation to the laboratory. From each sample, approximately 5 g of intestinal digesta was weighed and transferred into 10 mL of anaerobic FTG broth in a sterile filter-containing Whirl-Pak bag (Nasco, Fort Atkinson, WI, USA). The Whirl-Pak filter bag was stomached for 30 s (Stomacher 400 Circulator, Seward Limited, London, UK), after which 1 mL of the resulting filtrate was transferred into a test tube containing 9 mL of FTG broth for subsequent dilution and microbiological culturing to enumerate CP as described by Fasina et al. [11]. To enumerate CP colonies in each intestinal sample, the content of the test tube containing 1 mL of filtrate and 9 mL of FTG broth was subjected to a 5-fold serial dilution. The dilutions for each sample were then plated on blood agar (TSA with sheep blood) medium (Remel, Lenexa, KS, USA), using sterile plating beads (ZYMO Research, Irvine, CA, USA), and incubated anaerobically at 37 °C for 24 h. The number of characteristics round grayish CP colonies surrounded by a double zone of hemolysis was counted for each sample. The CP concentration was expressed as log_10_ CFU/g intestinal content.

Jejunum tissue samples for qRT-PCR were collected in DNase- and RNase-free cryovials, snap-frozen in liquid nitrogen, and stored at −80 °C until analysis. Blood was collected via heart puncture into tubes containing anticoagulant. The whole blood was centrifuged at 4200 rpm for 10 min using a Thermo Fisher Scientific centrifuge (Waltham, MA, USA). The resulting plasma was subsequently stored at −80 °C until time for subsequent analyses to determine biological antioxidant potential (BAP) and reactive oxygen metabolites (ROM).

#### 2.2.2. Lesion Scoring

The small intestine (Ileum, jejunum and duodenum) was assessed for intestinal lesions and evaluated on a score of 0–7, from no lesions and ulcers (0) to extensive lesions and ulcers (7), as described by [28].

### 2.3. Gene Expression Analysis

#### 2.3.1. RNA Isolation and Quantification

Jejunum tissue samples (30–40 mg) were processed for RNA extraction. Samples were weighed, cut using sterile tools, and transferred into 2 mL Sarstedt tubes containing 700 µL of lysis solution (Bio-Rad, Hercules, CA, USA) while kept on ice. Homogenization was performed with a bead-beater at 4800 rpm for 90 s, followed by centrifugation at 9000 rpm for 5 min at 4 °C. The supernatant was mixed with 700 µL of 60% ethanol and processed through an RNA binding column via sequential centrifugation and washing steps. DNA contamination was removed with 80 µL of DNase solution, incubated at room temperature for 25 min. After two wash steps, RNA was eluted with 80 µL of elution solution and centrifuged at 9000 rpm for 2 min. RNA purity and concentration were assessed at 260/280 nm using a BioTek Synergy H1 microplate reader (Bio-Rad, USA).

#### 2.3.2. Complementary DNA (cDNA) Synthesis and Quantitative Real-Time PCR (qRT-PCR) Analysis

cDNA synthesis was performed using the iScript Reverse Transcription Supermix (Bio-Rad, USA) based on RNA concentration. qRT-PCR was conducted with SYBR Green Supermix on the Bio-Rad CFX Connect system, using a reaction mix of cDNA, SSO Advanced Universal SYBR Supermix, primers, and DNase/RNase-free water. Reactions were run in triplicate on a sealed 96-well plate and optimized for a 60 °C annealing temperature. Gene expression was analyzed using the ΔΔCT method to determine relative fold changes in response to experimental conditions, as described by [29]. Quantitative real-time PCR was performed to evaluate the expression levels of proinflammatory cytokines; interleukin 1 beta (IL-1β), interferon gamma (IFN-ϒ), and tumor necrosis factor alpha (TNF-α), anti-inflammatory cytokines; interleukin 10 (IL-10), transforming growth factor beta (TGF-β), and protein-coding genes; and MUC2, which forms a protective mucus barrier in the small intestine. Glyceraldehyde 3-phosphate dehydrogenase (GAPDH) was considered a housekeeping gene in the jejunal tissues. Primers used for RT-PCR were designed in our laboratory using the National Center for Biotechnology Information (NCBI) Primer Blast. The primers used are listed in Table 2.

#### 2.3.3. Biological Antioxidant Potential and Reactive Oxygen Metabolites Assays

Biological antioxidant potential (BAP) and reactive oxygen metabolite (d-ROM) assays were conducted using the Free DUO test kit (DIACRON Research and Diagnostics, Grosseto, Italy). These assays were performed to evaluate oxidative status as described by Apalowo et al. [30].

For the d-ROMs assay, 20 µL of plasma and 20 µL of d-ROM reagent were sequentially pipetted into a thermally regulated cuvette using separate tips. The mixture was briefly stirred and then placed in the reading cell, where it was automatically incubated for 3 min, followed by a 2 min kinetic reading. For the BAP assay, 50 µL of BAP reagent was added to a thermally regulated cuvette, gently mixed, and placed in the reading cell for an initial 5 s reading. Subsequently, 10 µL of plasma was added to the cuvette, mixed gently, and incubated in the reading cell for 300 s [30].

### 2.4. Statistical Analysis

All experimental data were analyzed using one-way analysis of variance (SAS 9.4, Cary, NC, USA). Growth performance during the challenged phase was analyzed using a two-way ANOVA to evaluate the effects of diet (CON, GTX, GTY, and GTZ), CP challenge (CP 26 challenge versus no challenge) and the interaction effect of diet and CP26 challenge. The mean differences were determined using the Duncan multiple range test. This allowed the comparing of large mean pairs and protected against type II errors. Statistical significance was assumed at *p* ≤ 0.05.

## 3. Results

### 3.1. Growth Performance

Table 3 shows the effect of dietary GTE on growth performance of chicks from d 1–21. The result showed that unmedicated challenged control treatment (ACON) had the poorest (*p* ≤ 0.05) FCR of all treatments. Also, body weight gain and FCR were poorer in CP-challenged chicks compared to unchallenged chicks. All challenged GTE treatments showed similar FCRs to their corresponding unchallenged treatments, showing a propensity to ameliorate the effects of SNE in challenged birds. There were no differences in feed intake and overall mortality during this period.

Table 4 shows the effect of dietary GTE on growth performance during the CP challenge and SNE induction phase. The result shows that CP challenge significantly (*p* ≤ 0.05) affected weight gain and FCR, but did not influence feed intake which is characteristic of SNE disease. GTE supplementation significantly (*p* ≤ 0.05) influenced weight gain and feed intake but had no influence on FCR during this period. There were no interaction effects between CP challenge and dietary GTE levels across the board.

### 3.2. Intestinal Health Status

#### 3.2.1. CP Enumeration Data

Prior to challenge, intestinal CP colonization was significantly higher (*p* ≤ 0.05) in chicks fed the GTZ diet than in chicks fed the other (CON, GTX, and GTY) dietary treatments (Table 5).

Table 6 shows the results of intestinal CP on d 21 and that chicks fed GTZ diets had the highest (*p* ≤ 0.05) intestinal CP among unchallenged chicks at d 16. Unchallenged chicks fed CON and GTX diets had the lowest (*p* ≤ 0.05) intestinal CP compared to other treatments. Intestinal CP was generally higher (*p* ≤ 0.05) in CP challenge chicks than unchallenged chicks, showing a successful CP challenge. Chicks fed CON and GTX had similar intestinal CP after challenge which was significantly higher (*p* ≤ 0.05) than their corresponding unchallenged treatments, whereas chicks fed GTY and GTZ diets had similar intestinal CP in challenged chicks and also similar CP (*p* > 0.05) to their corresponding unchallenged chicks, which shows a prevention in establishing infection in these treatments (Table 6). Furthermore, challenged chicks fed GTX diets tended to have the lowest intestinal CP of all challenged treatments.

#### 3.2.2. Lesion Scores

No lesions were observed across all treatments.

#### 3.2.3. Gene Expression—Jejunum Tissue

**Figure 1:**
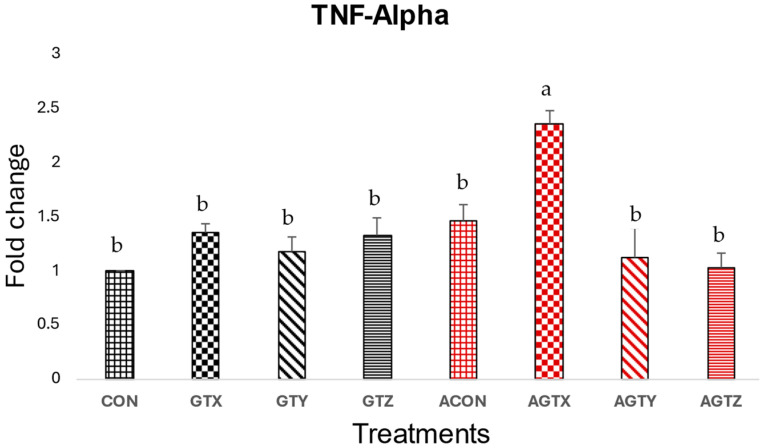
Effect of dietary GTE on Jejunal TNF-α expression (d 21). Challenge treatments have similar patterns as their corresponding unchallenged treatments but have a different (red) color. ^a–b^ means bearing different superscripts within a column are different (*p* ≤ 0.05).

Figure 1 shows a significantly higher (*p* ≤ 0.05) jejunal TNF-α expression in challenged chicks fed the GTX diet (AGTX) compared to those receiving other treatments. Challenged chicks fed GTY and GTZ (AGTY and AGTZ treatments) showed a tendency to lower TNF-α expressions compared to challenged treatments.

**Figure 2:**
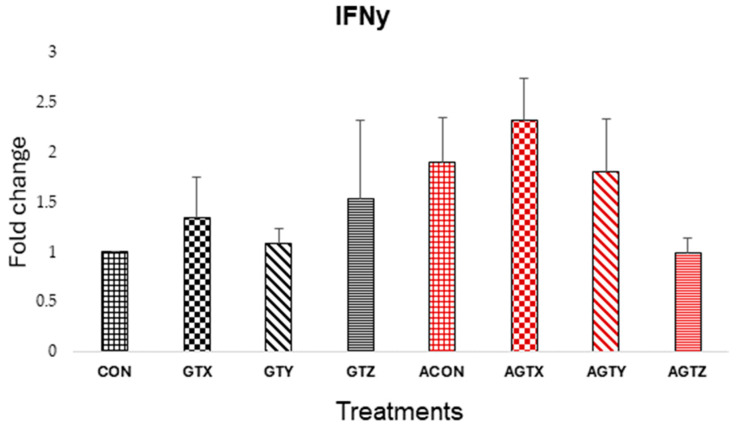
Effect of dietary GTE on Jejunal IFNγ expression (d 21). Challenge treatments have similar patterns as their corresponding unchallenged treatments but have a different (red) color.

There were no differences (*p* > 0.05) in jejunal IFNγ expressions (Figure 2); however, results showed that IFNγ expressions tend to be higher in challenged treatments, except in the AGTZ treatment, which showed a tendency to lower IFNγ compared to challenged treatments.

**Figure 3:**
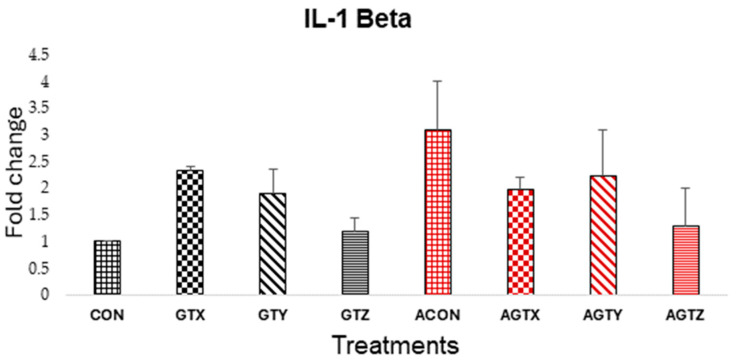
Effect of dietary GTE on Jejunal IL-1β expression (d 21). Challenge treatments have similar patterns as their corresponding unchallenged treatments but have a different (red) color.

While there were no differences (*p* > 0.05) in jejunal IL-1β expression (Figure 3), the CP-challenged chicks fed the control diet (ACON treatment) showed the highest expression level of all treatments, whereas GTE treatments showed a tendency to lower this inflammatory response in challenged chicks.

**Figure 4:**
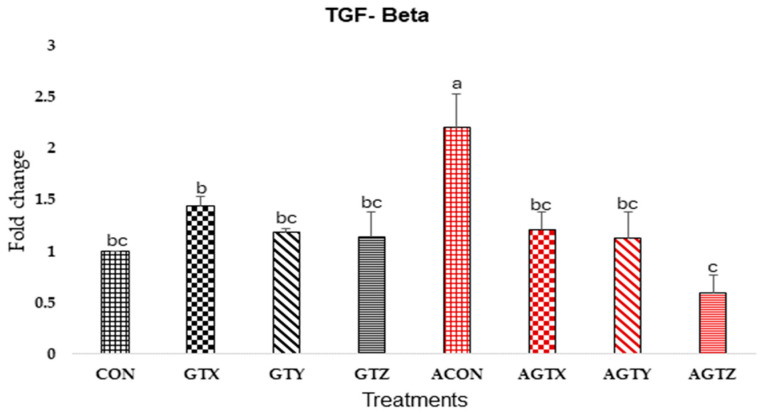
Effect of dietary GTE on Jejunal TGF-β expression (d 21). Challenge treatments have similar patterns as their corresponding unchallenged treatments but have a different (red) color. ^a–c^ means bearing different superscripts within a column are different (*p* ≤ 0.05).

Jejunal TGF-β expression (Figure 4) was the highest (*p* ≤ 0.05) in challenged control chicks (ACON). Challenged chicks fed GTZ diets (AGTZ treatment) showed the lowest (*p* ≤ 0.05) TGF-β expression, whereas AGTX and AGTY treatments showed similar TGF-β expressions to uncontrol treatments.

**Figure 5:**
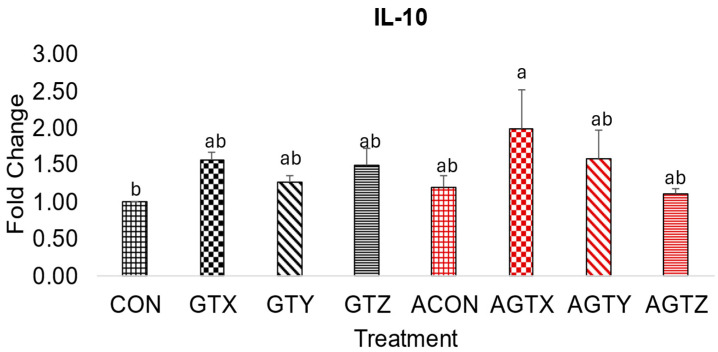
Effect of dietary GTE on Jejunal IL-10 expression (d 21). Challenge treatments have similar patterns as their corresponding unchallenged treatments but have a different (red) color. ^a–b^ means bearing different superscripts within a column are different (*p* ≤ 0.05).

IL-10 expressions (Figure 5) were the highest (*p* ≤ 0.05) in challenged chicks fed the GTX diet (AGTX treatment), showing a regulation of inflammatory and immune response in this challenged treatment.

**Figure 6:**
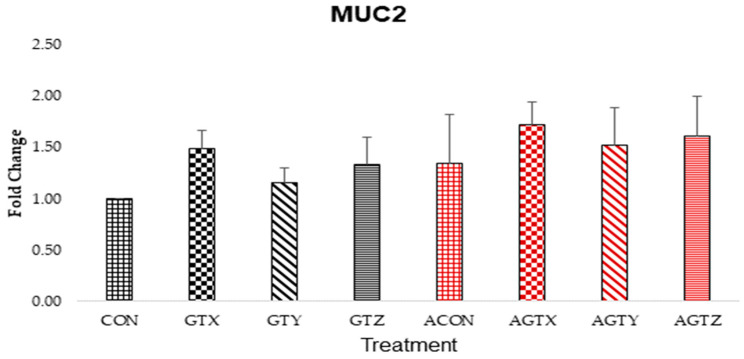
Effect of dietary GTE on Jejunal MUC-2 expression (d 21). Challenge treatments have similar patterns as their corresponding unchallenged treatments but have a different (red) color.

Jejunal MUC-2 expressions (Figure 6) showed a trend of increase (*p* > 0.05) in GTE treatments compared to the controls (with and without challenge).

**Figure 7:**
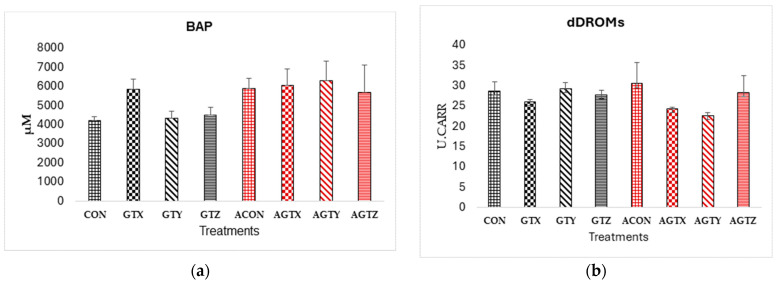
Effect of dietary GTE on oxidative status (d 21): (**a**) Biological antioxidant potential; (**b**) reactive oxygen metabolites. Challenge treatments have similar patterns as their corresponding unchallenged treatments but have a different (red) color.

There were no differences (*p* > 0.05) in biological antioxidant potentials and reactive oxygen metabolites across all treatments (Figure 7).

## 4. Discussion

The experimental induction of SNE was performed using dietary manipulation without co-infection. The basal diet was changed to a wheat and fish meal-based diet which has high protein and non-starch polysaccharides. With *CP* being an opportunistic pathogen, many researchers have relied on the use of *Eimeria* spp. co-infection to induce NE [31]. However, the successful induction of NE without co-infection was described by Shojadoost et al. [27]. In this study, experimental SNE was induced in chicks using multiple doses of CP challenge complemented by dietary manipulation.

The overall poor growth performance of CP-challenged birds, especially in the unmedicated control as compared to the unchallenged control treatment confirms the incidence of SNE, even when the subtle nature of SNE [2] meant no gross lesions and no differences were observed in mortality rate in the duration of the experiment (Table 3). The influence of CP challenge on growth performance during the challenge phase showing poorer weight gain and FCR compared to corresponding unchallenged treatments without affecting feed intake also establishes SNE incidence (Table 4). This result was similar to the findings of Zhang et al. [32] who evaluated the efficacy of four different plants extracts including pomegranate peel, *Sophora flavescens*, *Astragalus,* and *Artemisia annua* on growth performance of chickens with necrotic enteritis. During SNE, chicks may continue to feed without efficient conversion [27] due to subtle damage of the intestinal epithelia which may only be detected microscopically. Unlike clinical NE, which is characterized by severe intestinal lesion, growth depression, and high mortality, SNE is more subtle and difficult to diagnose [2,33].

In the context of GTE treatments, we found that the negative impact of SNE was seen ameliorated with GTE supplementation in challenged chicks as chicks fed on a GTE supplemented diet receiving the challenge had comparable growth performance to that of unchallenged control. GTE is known to have antimicrobial and growth promoting properties [18,19,22] which is seen to be beneficial to the chicks during SNE incidence.

With CP being a commensal in the gut [34,35,36], all the chickens irrespective of their treatments were found to have CP during the experiment (Table 5). However, challenged treatments generally showed higher intestinal CP counts reinforcing the CP challenge (Table 6). Although chicks fed higher levels of GTE showed higher intestinal CP count, these levels remained similar after being CP-challenged. Saeed et al. [22] reported that GTE has properties that promote growth of beneficial microorganisms, hence, it can be reasonably speculated that the higher levels of GTE may have promoted baseline CP, while preventing the clinical effects of pathogenic CP after challenge.

The expression levels of proinflammatory cytokines (TNF-α, IL-1β and IFN-γ), anti-inflammatory cytokines (TGF-β and IL-10) and a mucus protein-coding gene (MUC2) were evaluated in the study. The TNF-α, IL-1β, and IFN-γ are typically elevated during clinical NE and in some instances during SNE incidences as a result of an immune combat response to *C. perfringens* [37,38]. High levels of these cytokines contribute to inflammatory responses in the intestinal lining contributing to tissue damage and disruption of intestinal barrier function [31]. Although there were no statistically significant differences observed in IL-1β and IFN-γ expressions in the present study due to our SNE model features, a subtle numerical increase in the expression of these cytokine genes was evident in the unmedicated challenged (ACON) group, while chicks fed GTE supplementation above 250 mg/kg showed a numerical tendency to lower these inflammatory responses. These observations were somewhat similar to studies that investigated probiotic [39] or GTE effects resulting in reduced host inflammatory response [40]. Similarly, TNF-α expressions was lowered in chicks fed GTE supplementation above 250 mg/kg but highest in challenged chicks fed GTX, showing the potential of GTE to modulate inflammatory responses during SNE incidence.

During SNE incidence, TGF-β and IL-10 play important roles as anti-inflammatory cytokines can mitigate tissue damage caused by *Clostridium perfringens* [41,42,43]. This anti-inflammatory action is orchestrated by regulating immune response and promoting tissue repair causing the elevation of these cytokines in affected tissues of disease birds compared to healthy birds [28,43,44]. Our results showed that unmedicated challenged chicks (ACON) had the highest TGF-β expressions compared to all treatments confirming a modulated inflammatory response during CP challenge, possibly as a result of jejunal tissue damage. A numerical increase in the IL-10 anti-inflammatory response was seen in chicks fed the lowest GTE supplementation compared to other treatments further confirming the modulatory role of GTE during inflammation. Our gene expression results for IL-1β, IFN-γ, and IL-10 cytokines were similar to the findings of Song et al. [45], who fed dietary *Macleaya cordata* extract, another phytobiotic feed additive, to chickens with necrotic enteritis.

MUC2 mucin plays a role in the protection of gut barriers, the prevention of diseases, and the regulation of microbiome homeostasis [46]. GTE, being a polyphenolic compound, is a dietary component capable of modulating MUC2 expressions [46]. While there were no differences across all treatments, there was a trend of higher MUC2 expressions in chicks fed GTE supplementation especially when challenged, suggesting a propensity to combat bacterial infection in the intestine.

Biological antioxidant potentials and reactive oxygen metabolites are indicators of oxidative stress tested in the blood. They indicate the level of oxidative damage caused by free radicals, essentially providing an overview of the balance between antioxidants and oxidants in the system [47,48]. There were no differences in the BAP and dROMS results across all treatments, which suggests a balanced oxidative state and no oxidative damage in the system. This also might be due to the subclinical type of NE induced in the chicks.

## 5. Conclusions

In summary, the subtle nature of SNE ensures that there is no observable intestinal damage, however chicks challenged with CP had poorer performance than their corresponding unchallenged treatments, with dietary GTE supplementation showing a tendency to improve FCR in challenged chicks, abating the characteristic poorer FCR observed in SNE-induced chicks. Although baseline CP was higher in chicks fed higher GTE levels, proliferation and establishment of infection was prevented upon CP challenge in these chicks unlike the control treatment. Chicks fed GTE diets also showed a tendency to modulate inflammatory responses in jejunum tissues, favoring increased protective actions during SNE.

In conclusion, dietary GTE supplementation mitigated intestinal CP infection and could modulate inflammatory responses during SNE incidence in broiler chicks. Further studies are needed to determine the immunomodulatory effects of GTE during NE over a full broiler production cycle.

## Figures and Tables

**Table 1:** Composition of experimental diets ^1^.

Ingredients	Diet 1 (%, d 1–10)	Diet 2 (%, d 11–21)
Corn	60.93	-
Wheat	-	46.93
Soybean meal	29.87	35.95
Soy oil	3.58	3.99
Fish meal	-	9.99
Calcium carbonate	1.59	1.20
Monocalcium phosphate	1.79	0.20
Vitamin premix for broilers	1.00	1.00
Mineral premix for broilers	0.50	0.50
L-Lysine	0.35	0.10
DL-methionine	0.25	0.10
Threonine	0.14	0.40
**Calculated**		
% Crude protein	20	30

^1^ CON = no green tea extract supplementation; GTX = control diet supplemented with 0.025% green tea extract top-dressed; GTY = control diet supplemented with 0.050% green tea extract top-dressed; GTZ = control diet supplemented with 0.1% green tea extract top-dressed.

**Table 2:** Primer sequences for target genes and reference genes.

Gene	Forward Sequence (5′ to 3′)	Reverse Sequence (5′ to 3′)	Amplicon Size (Base Pairs)
GAPDH	GAA GCT TAC TGG AAT GGC TTT CC	CGG CAG GTC AGG TCA ACA A	198
TNF-α	CCC CTA CCC TGT CCC ACA A	TGA GTA CTG CGG AGG GTT CAT	150
TGF-β	TTC CCT GGT TTT TCC ATC AA	GTT ATT GTA TGC AGT CTG CTC	200
IFN-γ	AAAGATATCATGGACCTGGC	GGA GGT CAT AAG ATG CCA TT	241
IL1-β	CTG CAG AAG AAG CCT CG	TTG ATG TCG AAG ATG TCG AA	132
IL10	GTT CCA GGT CAA AGA GAG TT	TTC TTC AAA GTG CAG AGT GT	122
MUC2	ACG TGT GTG CCC ATC TCC AA	GGG GAC GCG TTG CAA TCA AA	200

**Table 3:** Effect of dietary GTE on growth performance (d 1–21).

Days	D 1–21				
Treatment	Body Weight	Body Weight Gain	Feed Intake	Feed Conversion Ratio	Mortality
CON	0.56 ^a^ ± 0.005	0.51 ^a^ ± 0.005	0.73 ± 0.01	1.43 ^c^ ± 0.03	0.25 ± 0.25
GTX	0.49 ^cd^ ± 0.007	0.45 ^bcd^ ± 0.007	0.68 ± 0.01	1.52 ^abc^ ± 0.01	0.00 ± 0.00
GTY	0.52 ^bc^ ± 0.007	0.47 ^bc^ ± 0.010	0.70 ± 0.01	1.48 ^bc^ ± 0.02	0.00 ± 0.00
GTZ	0.50 ^cd^ ± 0.012	0.46 ^bc^ ± 0.012	0.70 ± 0.02	1.55 ^abc^ ± 0.03	0.00 ± 0.00
ACON	0.54 ^ab^ ± 0.011	0.48 ^ab^ ± 0.012	0.81 ± 0.05	1.71 ^a^ ± 0.16	0.75 ± 0.48
AGTX	0.47 ^d^ ± 0.015	0.42 ^d^ ± 0.013	0.68 ± 0.03	1.62 ^abc^ ± 0.04	0.50 ± 0.29
AGTY	0.49 ^cd^ ± 0.007	0.44 ^cd^ ± 0.006	0.73 ± 0.02	1.65 ^ab^ ± 0.05	0.75 ± 0.48
AGTZ	0.50 ^cd^ ± 0.018	0.45 ^bcd^ ± 0.017	0.73 ± 0.05	1.61 ^abc^ ± 0.06	0.50 ± 0.50
*p* value	0.0002	0.0002	0.07	0.05	0.45

^a–d^ means bearing different superscripts within a column are different (*p* ≤ 0.05). CON: no green tea extract supplementation; GTX: control diet supplemented with 0.025% green tea extract top-dressed; GTY: control diet supplemented with 0.050% green tea extract top-dressed; GTZ: control diet supplemented with 0.1% Green tea extract top-dressed. ACON: CON + CP26 challenge; AGTX: GTX + CP26 challenge; AGTY: GTY + CP26 challenge; AGTZ: GTZ + CP26 challenge.

**Table 4:** Effect of dietary GTE on growth performance during CP challenge phase.

	Week 3 Only (D 14–21) Challenge Phase		
Treatment	Weight Gain	Feed Intake	Feed Conversion Ratio
CON	0.34 ^a^ ± 0.003	0.42 ± 0.006	1.25 ^d^ ± 0.025
GTX	0.29 ^b^ ± 0.003	0.39 ± 0.010	1.32 ^cd^ ± 0.024
GTY	0.31 ^b^ ± 0.05	0.40 ± 0.005	1.29 ^d^ ± 0.017
GTZ	0.31 ^b^ ± 0.009	0.40 ± 0.007	1.31 ^cd^ ± 0.015
ACON	0.30 ^b^ ± 0.012	0.46 ± 0.022	1.52 ^ab^ ± 0.118
AGTX	0.26 ^c^ ± 0.011	0.38 ± 0.023	1.47 ^abc^ ± 0.056
AGTY	0.28 ^bc^ ± 0.012	0.42 ± 0.019	1.55 ^a^ ± 0.081
AGTZ	0.28 ^bc^ ± 0.015	0.39 ± 0.032	1.37 ^bcd^ ± 0.043
*p* value	0.0001	0.0657	0.0037
**Sources of variation (2-way factorial)**			
CP challenge	<0.0001	0.594	<0.0001
Diet	0.0005	0.017	0.6328
Challenge × Diet	0.9068	0.394	0.2039

^a–d^ means bearing different superscripts within a column are different (*p* ≤ 0.05). CON: no green tea extract supplementation; GTX: control diet supplemented with 0.025% green tea extract top-dressed; GTY: control diet supplemented with 0.050% green tea extract top-dressed; GTZ: control diet supplemented with 0.1% green tea extract top-dressed. ACON: CON + CP26 challenge; AGTX: GTX + CP26 challenge; AGTY: GTY + CP26 challenge; AGTZ: GTZ + CP26 challenge.

**Table 5:** Effect of dietary GTE on intestinal CP pre-challenge (d 16).

Treatment	CP Log 10 CFU/g
CON	0.45 ^b^ ± 0.11
GTX	0.41 ^b^ ± 0.13
GTY	0.21 ^b^ ± 0.11
GTZ	1.64 ^a^ ± 0.34
*p* value	0.0001

^a–b^ means bearing different superscripts within a column are different (*p* ≤ 0.05). CON: no green tea extract supplementation; GTX: control diet supplemented with 0.025% green tea extract top-dressed; GTY: control diet supplemented with 0.050% green tea extract top-dressed; GTZ: control diet supplemented with 0.1% green tea extract top-dressed.

**Table 6:** Effect of dietary GTE on intestinal CP post-challenge (d 21).

Treatment	CP Log 10 CFU/g
CON	1.03 ^c^ ± 0.11
GTX	0.86 ^c^ ± 0.18
GTY	1.62 ^ab^ ± 0.09
GTZ	1.72 ^a^ ± 0.11
ACON	1.59 ^ab^ ± 0.21
AGTX	1.55 ^ab^ ± 0.30
AGTY	1.72 ^a^ ± 0.20
AGTZ	1.95 ^a^ ± 0.24
*p* value	0.006

^a–c^ means bearing different superscripts within a column are different (*p* ≤ 0.05). CON: no green tea extract supplementation; GTX: control diet supplemented with 0.025% green tea extract top-dressed; GTY: control diet supplemented with 0.050% green tea extract top-dressed; GTZ: control diet supplemented with 0.1% green tea extract top-dressed. ACON: CON + CP26 challenge; AGTX: GTX + CP26 challenge; AGTY: GTY + CP26 challenge; AGTZ: GTZ + CP26 challenge.

## Data Availability

Data will be made available on reasonable request.

## Abbreviations

The following abbreviations are used in this manuscript:

NE	Necrotic enteritis
SNE	Subclinical necrotic enteritis
AGP	Antimicrobial growth promoter
GTE	Green tea extract
CP	*Clostridium perfringens*
FCR	Feed conversion ratio
